# Identifying key environmental barriers experienced by persons with mild, moderate, or severe disability in Bankim Health District, Cameroon: a policy-targeted secondary analysis of data obtained with the World Bank and WHO model disability survey

**DOI:** 10.1186/s13690-021-00619-y

**Published:** 2021-06-07

**Authors:** Lindsay Lee, Ferdinand Mou, Alphonse Um Boock, Carolina Fellinghauer, Mirjam Kohls, Alarcos Cieza, Carla Sabariego

**Affiliations:** 1 Sensory Functions, Disability and Rehabilitation (SDR), World Health Organization, Geneva, Switzerland; 2 FAIRMED country office, Yaounde, Cameroon; 3 Institute for Medical Information Processing, Biometry and Epidemiology, LMU Munich, Munich, Germany; 4 Pettenkofer School of Public Health, Munich, Germany; 5 Department of Health Sciences and Medicine, University of Lucerne, Lucerne, Switzerland; 6 Swiss Paraplegic Research, Nottwil, Switzerland; 7 Center for Rehabilitation in Global Health Systems, WHO Collaborating Center, University of Lucerne, Lucerne, Switzerland

**Keywords:** Cameroon, Disability, Functioning, Health policies, Public health, Random Forest, Statistical analysis

## Abstract

**Background:**

Comprehensive data is key for evidence-informed policy aiming to improve the lives of persons experiencing different levels of disability. The objective of this paper was to identify the environmental barriers — including physical, social, attitudinal, and political barriers — that might become priorities for cross-cutting policies and policies tailored to the needs of persons experiencing severe disability in Cameroon.

**Methods:**

A secondary analysis of data obtained with the WHO Model Disability Survey was completed in the Bankim Health District (*N* = 559) using random forest regression to determine and compare the impact of the environmental factors on the experience of disability.

**Results:**

The physical environment had by far the highest influence on disability, with transportation, toilet of the dwelling, and the dwelling itself being the most important factors. Factors inside one’s own home (toilet of the dwelling, and the dwelling itself) were the most important for persons with moderate and severe disability, followed by attitudes of others and issues with accessing health care.

**Conclusion:**

Our study provides country policy makers with evidence for setting priorities and for the development of evidence-informed policies for the Bankim Health District in Cameroon.

## Background

### The United Nations’ 2030 agenda for sustainable development includes disability as a central priority in five of the 17 sustainable development goals (SDGs)

In the spirit of “leaving no one behind” [1], the SDGs recognize persons with disability in the areas related to education, economic growth, reducing inequality, sustainability of cities and communities, and availability of data. As a consequence, efforts have been made to strengthen high-quality data collection in the field of disability and to enhance the availability of data that is disaggregated by disability, as reported by the UN Statistical Commission in the forty-ninth session in the UN Economic and Social Council [2].

### To achieve SDGs milestones, it is important to identify which interventions need to be implemented in countries and communities

Countries that are developing disability policies are particularly interested in understanding which issues are most relevant for persons with disability and which actions need to be taken. Comprehensive disability data can meaningfully contribute to the effective development of targeted disability policy by defining priorities at the micro-, meso- and macro-level.

### WHO currently supports the collection of disability data through the WHO and World Bank model disability survey (MDS)

The MDS is grounded in the International Classification of Functioning, Disability, and Health (ICF) [3], and operationalizes disability as a universal experience that lies on a continuum ranging from no to extreme disability [4]. In line with the ICF, disability is defined as the outcome of the interaction between health conditions (including diseases, congenital malformations, blindness, deafness, and loss of health due to the aging process) and the context of a person. The ICF describes the environmental context as a determinant (together with health conditions) of disability. Environmental factors are extrinsic to the individual and include, but are not limited to: the natural and human-made changes to the environment (rural versus urban settings); the climate (for instance, very dry or mountain regions), the social context (for instance, having or not having family support or support of friends); the attitudinal context (for instance being treated with respect by neighbours or health professionals or not); the health system (for instance having access to health services or whether health services are at all available).

The MDS was developed based on three key principles [4]. Firstly, that disability is a universal experience that can affect anyone over the course of life. Because of this principle, the MDS uses a representative sample of the complete population of a country or region, and is not restricted to specific groups with important impairments. Secondly, that disability is not a direct consequence of an underlying health condition or impairment but the outcome of its interaction with the context of a person. Consequently, the MDS includes questions about a range of environmental factors, going far beyond the usual focus of disability surveys on assistive technologies. Finally, the MDS operationalizes the principle that disability is not a dichotomous characteristic of a person (i.e., disabled versus not disabled) but an experience that lies on a continuum and ranges from no to extreme disability. Cut-offs are used to identify four groups: people who experience no, mild, moderate, or severe disability. These groups are compared across a large range of indicators. The comparison between persons with severe disability and persons with no disability is of utmost importance for monitoring the Convention on the Rights of Persons with Disabilities (CRPD) and specific SDG indicators.

For measurement purposes, two concepts are used in the MDS to operationalize disability: capacity and performance [4]. Capacity is the synthesis of a person’s intrinsic physical and mental capacities, determined by their health conditions or impairments and personal characteristics. Performance represents the outcome of the interaction between the individual’s capacity and features of the physical, human-made, attitudinal, and socio-political environment in which the person lives and corresponds to the conceptualization of disability in the ICF. Therefore, when reporting disability using the MDS, the performance metric is considered.

The MDS was implemented as a regional survey in the Bankim Health District, Adamawa region, Cameroon in 2016 to shed light on the situation of persons with disability living in this region and to guide the identification of intervention targets. The implementation of the MDS in Cameroon meets calls for efforts in the investigation of the life situations of persons with disability in Cameroon [5]. The survey was carried out in all seven health areas of the district with a general and representative population sample. The specific objectives of the MDS implementation were (1) to estimate the prevalence of disability, (2) to determine the disability distribution, (3) to identify needs, barriers and inequalities faced by persons with different levels of disability, and (4) to provide the information necessary for the development of future evidence-informed policy priorities in order to improve the lives of persons with disability in the Bankim Health District.

In the present secondary analyses, we used data from the MDS implementation in Cameroon to address the fourth objective described above. Our overall objective was to identify the environmental factors — including hindering and facilitating factors of the physical, social, attitudinal and political environment — that might become priorities for cross-cutting policies and for policies tailored to the needs of persons experiencing severe disability in Cameroon. Specifically, we aim to answer the following questions:

1. Which factors of the environment are most relevant for persons with mild, moderate, or severe levels of disability?
2. To what extent does the relevance of environmental factors change depending on the level of disability?
3. Which aspects of the environment should become priorities for disability policies?

It is important to stress that in response to emerging needs for research in the context of ongoing developments, we used an approach to data analysis that goes beyond the usual disaggregation of information by disability status and instead focuses on the study of environmental factors from a broad perspective. Based on the comprehensive information collected with the MDS, we established a ranking of the relative importance of various environmental factors for persons with different levels of disability. Policy makers working in Cameroon, a country in which persons with disability face a range of barriers – such as the lack of public transportation, stigma, poor health care or few employment opportunities – might be overwhelmed by the breadth of problems faced and challenged by restricted human and financial resources. By providing a ranking of the most hindering barriers experienced by persons with mild, moderate, or severe disability, we provide stakeholders with the evidence needed for prioritization of policies and public health investments in light of limited resources.

## Methods

The original data collection and implementation of the MDS in the Bankim Health District, Cameroon was carried out by WHO in two phases. After a pretest in December 2015, the full implementation took place during the first half of 2016 with 559 interviews. The present study is a secondary data analysis performed between April and July 2018 using the data collected through individual interviews. In this secondary analysis, we followed a two-step approach. In the first step, we identified which type of environmental factors – physical environment, social support, social network, attitudinal environment, access to information, medication, personal assistance, assistive devices, and responsiveness of health care system – were the most relevant for the whole sample overall as well as for specific groups, namely persons with moderate, severe or mild disability. In a second step, we identified which specific aspects of three types of environmental factors – physical environment, attitudinal environment, and responsiveness of health care system – were the most relevant for the whole sample as well as for persons with moderate, severe or mild disability. This approach should provide stakeholders with precise information about which areas to target to improve the lives of persons with disability.

### Study design, participants and region

The WHO MDS was conducted in the Bankim Health District of the Adamawa region in Cameroon in 2016. The objectives of the implementation of the MDS were to estimate the prevalence of disability and its current distribution in the Cameroonian population in the Bankim Health District and to identify needs, barriers and inequalities faced by persons with different levels of disability. The survey comprises a household questionnaire and an individual questionnaire completed by a random adult (aged 16+) member of the household. The individual questionnaire includes sections on (1) socio-demographic characteristics, (2) work history and benefits, (3) environmental factors, (4) functioning, (5) health conditions and capacity, (6) health care utilization and (7) satisfaction, personality, and well-being.

The questionnaires and further information on the MDS are available at http://www.who.int/disabilities/data/mds/en/.

The population of the Adamawa region at the time of the survey was 1,205,861 inhabitants. An estimated 97,790 inhabitants lived in the 2700 km^2^ of the Bankim health district in 2015 [6]. Bankim has a hot and equatorial climate. Reported main economic activities are livestock breeding, farming, fishery, trade, craftsmanship, and motorbike taxis. The district has several resources, including gallery forests, dense forests, arable land for food and cash crops, tourist sites, shallows, swamps, lakes, rivers, and sand. However, this potential remains mostly unexploited. The Communal Plan of the Bankim District from 2015 [6] reports that the district’s infrastructure is insufficient to guarantee healthy living for the population. For instance, water supplies are frequently contaminated, and the villages’ electrification and sewage disposal channels, are insufficient. There is a lack of a communal plan for household management and few poorly maintained roads. The health care situation is precarious, with insufficient professionals and insufficient infrastructure. In 2015, the health facilities available in the Bankim district consisted of one District Hospital, one District Medical Center, and 12 Integrated Health Care Facilities employing a total of 3 medical doctors, 7 qualified nurses, and 21 caregivers. Some of the Integrated Care Facilities function without electricity, beds, or water points.

Information about disability has been collected in the 2010 census and in the Multiple Indicator Cluster Survey [7] in 2011. However, it is important to note that these surveys understood disability from a much narrower perspective as the one used in the Model Disability Survey and generally focused solely on persons with severe impairments, mostly on persons who are blind, deaf, or that cannot walk. The 2010 census estimated nearly 3 million people in the country are disabled, and the disability rate published by the MICS [7] was 5.4% in 2011. An overview of the measurement of disability in Cameroon can be found in Simo Fotso et al (2019) [8]. The MDS, implemented in the Bankim health district in 2016, uses a much broader and inclusive approach to measuring disability and estimated a rate of severe disability for that region of 13.2%. This group most commonly reported severe problems with their mobility, including problems walking and standing. It is important to stress that MDS results refer to the health district of Bankim and are not expected to be representative of the many regions of Cameroon.

### Variables

#### Performance

Performance was the dependent variable in our study. It describes the level of disability experienced by a person and is defined as how a person performs in daily life given their health state as well as the different facilitating or hindering aspects of their environment. The questions of Module 4000 of the MDS assess the extent to which a person experiences problems in multiple domains of functioning. The information from this module was combined to create a single performance score ranging from 0 (no problems in performance) to 100 (extreme problems in performance) using Polytomous Rasch Analysis [9, 10] in the scope of the original data analyses conducted by WHO in 2018. The Rasch analysis allows testing a scale’s measurement properties and primarily ensures that the sum score is usable as an interval-scaled metric. The data analysis strategy used in the MDS, including details of the Rasch analyses, is reported elsewhere [11].

Groups with different disability levels were determined based on the distribution of the performance score. Persons with scores lower than the mean minus one standard deviation (SD) are considered to have no disability; persons with scores higher than mean minus one SD but lower than the mean are considered to have mild disability; persons with scores higher than mean but lower than the mean plus one SD are considered to have moderate disability; persons with scores higher than the mean plus one SD are considered to have severe disability.

#### Environmental factors

Predictor variables were included to capture the multiple dimensions of environmental factors, namely the physical environment, social support, social network, attitudinal environment, access to information, medication, personal assistance, assistive devices, and responsiveness of the health care system. The entire list of environmental factors included in the analysis are found in Annex 1.

The variable capturing the participants’ social network size was created by adding up the total number of close relationships among family, friends, co-workers, and neighbors. The network size represented a 5-level ordinal variable with categories grouping 0, 1, 2–4, 5–14, or 15+ close persons. Relationships were understood as close if one could, for instance, talk about personal affairs, get help, or enjoy spending leisure time with the other person. The MDS assessed the access to information with the question “Do you have access to the information you need or want?” with five response options ranging from “not at all” to “yes completely”. The regular intake of medication for the treatment of chronic disease was assessed with the binary question, “Do you take medicines on a regular basis?”. Information on personal assistance was summarized in a variable categorizing “*use of personal assistance and additional need,” “use of personal assistance and no additional need,” “no use but need for personal assistance,”* and *“no use and no need for personal assistance*.” Information on assistive devices in the areas of mobility, seeing, hearing, work, education, home, or community was aggregated and categorized in the same way as for personal assistance.

Polytomous Rasch Analysis was used to create metric scores with a range from 0 to 100 for the variables describing hindering or facilitating physical environment, social support, attitudinal environment, and responsiveness of health care system in the scope of the original data analysis carried out by WHO.

#### Control variables

Age, sex, education, work, and capacity were included in the analysis as control variables. Educational level and work status served as proxy for socio-economic status. Capacity refers to a person’s health state. It is measured in Module 5000 of the MDS and targets the difficulties a person may have in his or her life due to health without considering environmental barriers and facilitators. Disability is defined as the result of the interaction between a person’s health and their environment. Therefore, the analysis was adjusted for capacity to obtain an unbiased estimate of the impact of the different environmental factors on disability. The capacity measure was operationalized using a metrical scale created with Polytomous Rasch Analysis in the scope of the original data analyses.

### Statistical analyses

We used random forest regression to determine and compare the impact of the environmental factors on the experience of disability. Random forests are statistical learning methods that are based on decision trees [12]. An ensemble of de-correlated trees is generated by training many single decision trees on bootstrap sub-samples of the original data using a random subset of the predictors. The final random forest is achieved by averaging the collection of trees, reducing the variance of the model.

The model fit of the random forest was assessed using the root mean square error (RMSE) as measure of prediction accuracy. The test set for measuring the prediction error is composed of the part of the observations that is not used in the training of the trees due to the bootstrap sub-sampling, called out-of-bag observations (OOB).

We derived the permutation variable importance (VIM) from the random forest models to serve as a measure of the association between the predictors and performance score and used it to create a ranking of importance of the predictors. It is defined as the mean decrease in prediction accuracy after permutation of the predictor variable in the OOB test set, quantifying how much a given variable adds to the accuracy of the model. The mean square error can serve as reference against which to compare the VIM.

The first random forest model was built to include the variables physical environment, social support, social network, attitudinal environment, access to information, medication, personal assistance, assistive devices, and responsiveness of the health care system. Further models included the single questions of the MDS related to physical environment, attitudinal environment, and responsiveness of health care system, respectively. Annex 1 contains the survey questions for each of these areas and their MDS-codes.

The models were applied to both the total sample and groups with low or high levels of disability. For this purpose, we divided the sample into no or mild disability and moderate or severe disability. The threshold for the cut-off between these two groups was the sample mean of the performance score.

Analyses were run in R v3.4.4. The R function cforest from the R package party v1.3–0 was used to perform the random forest regression [13, 14]. The R package caret v6.0–79 was used for calculating the RMSE and VIM [15]. The number of trees for all models was set to 5000, after checking for sufficient convergence of the prediction accuracy; the number of predictors for each tree was set to 5.

## Results

### Characteristics of the study population

The performance score, the dependent variable in the present study, had a mean of 40.2 in the total sample (Table 1). As reported in the methods section, the performance score ranged from 0 to 100, where 0 indicated no disability and 100 the highest level of disability. Applying the cut-offs to the distribution obtained for the Bankim Health District, persons with scores below 25.83 experience no disability, persons with scores between 25.83 and 38.99 experience mild disability, persons with scores between 38.99 and 52.15 experience moderate disability, and persons with scores above 52.15 experience severe disability. Since the mean performance score for the complete sample is 40.2 (Table 1), the average of the surveyed population in the Bankim Health district lies near the lower bound for a moderate level of disability.

**Table 1:** Sociodemographic characteristics of the study population. Data Source: Model Disability Survey, Bankim Health District, Cameroon, 2016

		Total sample (***N*** = 559)		Moderate and severe disability (***N*** = 312)		No and mild disability (***N*** = 247)	
		%	(N)	%	(N)	%	(N)
**Gender**	Male	49.4	(276)	46.8	(146)	52.6	(130)
	Female	50.6	(283)	53.2	(166)	47.4	(117)
**Age**	16–39	53.7	(300)	45.2	(141)	64.4	(159)
	40–59	29.0	(162)	31.1	(97)	26.3	(65)
	60+	14.3	(80)	19.6	(61)	7.7	(19)
**Work status**	Not working	42.8	(239)	45.8	(143)	38.9	(96)
	Working	57.2	(320)	54.2	(169)	61.1	(151)
**Level of education**	No education	20.9	(117)	27.9	(87)	12.1	(30)
	Elementary	33.8	(189)	35.9	(112)	31.2	(77)
	Vocational	17.4	(97)	17.0	(53)	17.8	(44)
	Secondary	17.2	(96)	11.2	(35)	24.7	(61)
	Higher	7.0	(39)	5.4	(17)	8.9	(22)
	Other	0.5	(3)	0.6	(2)	0.4	(1)
		**Mean**	**(SD)**	**Mean**	**(SD)**	**Mean**	**(SD)**
**Performance score (0–100)**		40.2	(12.4)	47.5	(7.3)	30.9	(11.3)
**Capacity score (0–100)**		29.2	(20.9)	44	(14.7)	10.6	(9.6)

The gender distribution in the sample was fairly even. Most of the sample (50.6%) belonged to the age group 16–39 years.

### Impact of environmental factors on performance

Table 2 shows the most important environmental factors. The five environmental factors with the highest impact among the total sample were the physical environment, social support, assistive devices, personal assistance, and access to information. However, it is important to note that the VIM size indicated that the physical environment (25.49) had a much larger effect on performance than social support (9.2). There were overlaps for persons with high and low levels of disability, but some important differences were observed: for persons with moderate and severe disability, the attitudinal environment was among the three most highly ranked variables while access to information was the least highly ranked factor. For persons with no and mild disability, the social support had a higher VIM than the physical environment.

**Table 2:** Results of the random forest showing the permutation variable importance measure (VIM) and ranking for the environmental predictors of performance. Data Source: Model Disability Survey, Bankim Health District, Cameroon, 2016

	MDS-Code	Variable	Total sample		Moderate and severe disability		No and mild disability	
			VIM	Rank	VIM	Rank	VIM	Rank
**Environmental factor**	**I3001 - I3010**	Physical environment	25.49	1	9.2	1	6.87	2
	**I3017 - I3019**	Social support	9.2	2	1.41	2	16.05	1
	**I3040 - I3070**	Assistive devices	2.88	3	0.65	4	3.74	3
	**I3017-I3020**	Personal assistance	1.14	4	0.33	5	0.86	5
	**I3035**	Access to information	0.98	5	0.03	9	2.03	4
	**I6021 - I6027**	Health care system	0.94	6	0.15	6	0.73	6
	**I3024 - I3034**	Attitudinal environment	0.78	7	0.75	3	0.72	7
	**I3021 - I3023**	Social network	0.26	8	0.05	7	0.2	8
	**I3013**	Medication	0.13	9	0.04	8	0	9
**Control variable**		Capacity	87.42		24.24		26.09	
		Age	3.17		1.38		1.86	
		Gender	0.02		0		−0.05	
		Education	0.14		0.13		−0.32	
		Work	0.66		0.44		0.26	
		**RMSE**	7.43		5.08		9.46	

#### Physical environment

The physical environment is one of the most relevant environmental factors across disability (Table 2). The analysis of specific aspects of the physical environment shows that their importance vary considerably when the level of disability is taken into account (Table 3). The five aspects with the highest impact among the total sample are transportation, the toilet in one’s dwelling, the dwelling itself, lighting, noise, and crowds, as well as temperature, terrain, and climate. While toilet in one’s dwelling, the dwelling itself and places to worship are the most important aspects for persons with moderate and severe disability, the most important ones for persons with no and mild disability are transportation, lighting, noise and crowds, and workplace or education institution.

**Table 3:** Results of the random forest showing the permutation variable importance measure (VIM) and ranking for aspects the physical environment as predictors of performance. Data Source: Model Disability Survey, Bankim Health District, Cameroon, 2016

	MDS-Code	Variable	Total sample		Moderate and severe disability		No and mild disability	
			VIM	Rank	VIM	Rank	VIM	Rank
**Environmental factor: physical environment**	**I3006**	Transportation	5.46	1	0.64	5	4.89	1
	**I3008**	Toilet of your dwelling	5.4	2	4.52	1	0.03	8
	**I3007**	Dwelling	3.62	3	3.44	2	0.49	4
	**I3010**	Lighting, noise, crowds	2.79	4	0.16	8	1.86	2
	**I3009**	Temperature, terrain, climate	2.31	5	0.93	4	0.13	7
	**I3001**	Workplace or education institution	1.94	6	0.06	9	1.4	3
	**I3003**	Places for socializing and community activities	1.8	7	0.34	6	−0.06	10
	**I3005**	Places of worship	1.68	8	1.45	3	0.15	5
	**I3002**	Health facilities	0.82	9	0.02	10	0.02	9
	**I3004**	Shop, banks, post office	0.71	10	0.24	7	0.15	6
**Control variable**		Capacity	82.4		21.91		29.06	
		Age	2.62		1.51		1.89	
		Gender	−0.09		−0.01		− 0.43	
		Education	− 0.06		0.15		−1.63	
		Work	0.78		0.34		0.49	
		**RMSE**	7.85		5.1		10.16	

#### Attitudinal environment

The five aspects of the attitudinal environment with the highest VIM for the total sample were problems getting in involved in society, considering yourself a burden on society, feeling accepted by others, living with dignity, and feeling respected by others (Table 4). When stratifying by the level of disability, considering oneself a burden on society, feeling respected by others, and living with dignity become the three most essential aspects for persons with moderate and severe disability. The three most important aspects for persons with no and mild disability were feeling accepted by others, feeling respected by others, and people around you tending to become impatient with you.

**Table 4:** Results of the random forest showing the permutation variable importance measure (VIM) and ranking for aspects the attitudinal environment as predictors of performance. Data Source: Model Disability Survey, Bankim Health District, Cameroon, 2016

	MDS-Codes	Variable	Total sample		Moderate and severe disability		No and mild disability	
			VIM	Rank	VIM	Rank	VIM	Rank
**Environmental factor: attitudinal environment**	**I3025**	Do you have problems getting involved in society because of the attitudes of people around you	3.91	1	−0.02	11	1.47	7
	**I3031**	Do you consider yourself a burden on society	2.74	2	2.67	1	0.51	9
	**I3029**	Do you feel that other people accept you	1.96	3	0.67	4	5.5	1
	**I3034**	Is living with dignity a problem for you because of the attitudes and actions of others	1.8	4	0.72	3	0.37	10
	**I3030**	Do you feel that other people respect you	1.63	5	1.49	2	2.79	2
	**I3028**	Do you get to make the big decisions in your life	1.08	6	0.32	5	1.93	4
	**I3027**	Do you make your own choices about your day-to-day life	0.98	7	0.21	6	1.81	6
	**I3033**	Do people around you not expect much from you	0.97	8	0.06	10	1.83	5
	**I3024**	Can you participate in family decisions	0.89	9	0.07	9	1.36	8
	**I3026**	Do you feel that some people treat you unfairly	0.87	10	0.14	8	0.29	11
	**I3032**	Do people around you tend to become impatient with you	0.8	11	0.15	7	2.6	3
**Control variable**		Capacity	113.73		27.26		27.76	
		Age	7.42		2.43		1.68	
		Gender	0.12		−0.02		0.2	
		Education	0.36		0.13		−1.02	
		Work	0.86		0.92		−0.21	
		**RMSE**	8.07		5.35		9.88	

#### Responsiveness of health care system

Table 5 shows results for specific aspects of the responsiveness of the health care system. The most important variables for the total sample were the waiting time before being attended to, the ease with which you could see a health care provider you were happy with, and the experience of being involved in making decision for your treatment. The ranking differed considerably for persons with high and low levels of disability. The aspects that matter most for persons with moderate and severe disability were the experience of being treated respectfully, how clearly health care providers explained things to you, and the experience of being involved in making decisions for your treatment. For persons with no and mild disability, the most important aspects were the waiting time before being attended to, the cleanliness of the health facility, and the ease with which you could see a health care provider you were happy with.

**Table 5:** Results of the random forest showing the permutation variable importance measure (VIM) and ranking for aspects the responsiveness of the health care system as predictors of performance. Data Source: Model Disability Survey, Bankim Health District, Cameroon, 2016

	MDS-Codes	Variable	Total sample		Moderate and severe disability		No and mild disability	
			VIM	Rank	VIM	Rank	VIM	Rank
**Environmental factor:** **attitudinal environment**	**I3025**	Do you have problems getting involved in society because of the attitudes of people around you	3.91	1	−0.02	11	1.47	7
	**I3031**	Do you consider yourself a burden on society	2.74	2	2.67	1	0.51	9
	**I3029**	Do you feel that other people accept you	1.96	3	0.67	4	5.5	1
	**I3034**	Is living with dignity a problem for you because of the attitudes and actions of others	1.8	4	0.72	3	0.37	10
	**I3030**	Do you feel that other people respect you	1.63	5	1.49	2	2.79	2
	**I3028**	Do you get to make the big decisions in your life	1.08	6	0.32	5	1.93	4
	**I3027**	Do you make your own choices about your day-to-day life	0.98	7	0.21	6	1.81	6
	**I3033**	Do people around you not expect much from you	0.97	8	0.06	10	1.83	5
	**I3024**	Can you participate in family decisions	0.89	9	0.07	9	1.36	8
	**I3026**	Do you feel that some people treat you unfairly	0.87	10	0.14	8	0.29	11
	**I3032**	Do people around you tend to become impatient with you	0.8	11	0.15	7	2.6	3
**Control variable**		Capacity	113.73		27.26		27.76	
		Age	7.42		2.43		1.68	
		Gender	0.12		−0.02		0.2	
		Education	0.36		0.13		−1.02	
		Work	0.86		0.92		−0.21	
		**RMSE**	8.07		5.35		9.88	

## Discussion

Using data from a regionally representative implementation of the World Bank and WHO Model Disability Survey in the Bankim Health District, Adamawa, Cameroon, allowed identifying which environmental factors were the most relevant ones for persons with different levels of disability. Among the whole population, the physical environment had by far the highest impact on disability, with transportation, toilet of the dwelling, and the dwelling itself being the most important aspects. Aspects inside one’s own home were the most important for persons with moderate and severe disability, indicating that for this group accessibility improvements in the public sphere will likely have little effect until the barriers in their homes are addressed.

### Our results must be understood in light of the context of the Bankim health district

The district is poor, and the infrastructure is deficient in several aspects. There is no public transportation, and private cars are expensive, so the areas outside of Bankim are only accessible by motorbike or only during the dry season. This considerably hinders access to health facilities, especially for persons with mobility problems. Our results reflect the negative impact of inexistent public transportation on the district’s population, especially on persons with moderate to severe disability. The communal development plan of the commune of Bankim from 2015 [6] states that access to adequate housing in Bankim needs to be improved. The construction of dwellings was described as uncontrolled, lacking compliance with construction standards and corresponding regulations. Insufficiency of appropriate latrines and water points was reported as well. Progress has been made regarding sanitation in the urban area. Nevertheless, the inadequacy of wastewater drainage channels, and the absence of a structure in charge of emptying out-of-use septic tanks remain major issues. Consistently, our results confirm the burden posed by the lack of a toilet in the dwelling, and the construction of the dwelling itself, on the daily life of persons with moderate to severe disability.

### Environmental factors are by definition context-specific, so differences in results from similar studies carried out in different countries or regions are expected

For instance, a study using data from a Spanish Survey on Disability including 17,303 adults identified discrimination, economic benefits due to disability and support from family and friends as the most important predictors of disability levels [7]. Two further studies have used data collected with the MDS in Chile and Cambodia to study the impact of environmental factors on disability. In a pilot study in Cambodia, places to socialize for community activities, transportation, and the natural environment as well as use and need of personal assistance and use of medication on a regular basis were the most important cross-cutting environmental factors [16]. A large representative study in Chile aiming to identify which environmental factors were the most responsible for disability experienced by persons with mental disorders identified the availability and frequency of personal assistance and assistive devices for mobility as the most important environmental factors across conditions, but discrimination and use of health services also played an important role [17]. The district we researched in Cameroon has poor infrastructure and our results indicate that major barriers are the lack of public transportation and the building of the dwellings, including toilets. All these examples reiterate the importance of collecting detailed data on disability for guiding a meaningful and context-relevant evidence-informed implementation of the CRPD.

### Our study clearly demonstrated that going beyond typical dichotomous measurement of disability (disabled x not disabled) and measuring disability as a matter of degree, ranging from low to high levels, is key for evidence-informed policy making

In Cameroon, there are meaningful differences in the environmental factors that were the most important and had the highest impact on disability across different individual disability levels. For instance, the attitudinal environment, especially not feeling as if one is a burden on society and living with dignity, were much more important for persons with moderate and severe disability than for those with no or mild disability. This suggested that social stigma against persons with disability in Cameroon has a disproportionate impact on those who experienced the most severe levels of disability. In order to reach the most vulnerable in the Adamawa region, policy makers must invest not only in increasing accessibility of dwellings but also in changing society’s mindset towards disability. Dedicated disability surveys, as the MDS, which are representative, focus on levels of disability and include a comprehensive inventory of environmental factors that impact disability (not only assistive technologies) are essential to perform situation analyses that can support a meaningful implementation of the CRPD.

### The WHO global disability action plan has set as an objective to improve the access to health services for persons with disability

Responsiveness of the health care system is an environmental factor and was generally ranked positively across disability levels in the Bankim region. However, there are large differences within this factor. Domains related to convenience (e.g., time you waited, cleanliness, ease of seeing a provider you are happy with) were the most important factors for persons with no and mild disability. However, for persons with moderate and severe disability, domains related to fundamental rights (e.g., being treated respectfully, having things explained clearly by health care providers, and being involved in making treatment decisions) were the most important ones. This indicates that as Cameroon works to improve its health care system, it is important to ensure that basic rights of all health care recipients, including persons with severe disability, are respected. These results were in line with published literature in other settings [18, 19].

### The richness of evidence obtained in this study raises the question of how to move forward having now identified highly relevant targets for interventions

In a next step, policy makers must come together with other stakeholders to define priorities, suitable interventions, and timeframes, taking into account the cultural, political, and economic reality of this region. WHO has supported countries with policy dialogues, particularly in the African region, through the preparation of detailed policy briefs [20]. The results of the present study can serve as basis for the preparation of accessible policy briefs. Additionally, implementation research, defined by WHO as “the scientific study of methods to promote the systematic uptake of research findings and other evidence-based practices into routine practice”, offers the tools necessary to support the development, sustainable implementation and evaluation of public health and policy changes [21].

The present study must also be understood in light of its limitations. Firstly, the sample size was calculated as the minimum size needed to represent the district while still maintaining a low budget. The sample size was estimated using Lorenz’s sample size determination formula for cross-section studies, and a minimum of 545 households was required. The individual within the household was selected randomly. The low sample size made it possible to implement the survey in the Bankim health district, but it does not allow for high levels of disaggregation. Secondly, the health district of Bankim and the region of Adamawa are very particular; while presented results can be generalizable to some similar regions, they do not reflect Cameroon’s situation in general. Nevertheless, the results of this study are essential and substantive in the light of the concerning lack of information on disability in Cameroon.

## Conclusion

Our study provides stakeholders in Cameroon with specific information for the prioritization of intervention areas and the development of evidence-informed policies. It especially shows the importance of going beyond disaggregation, not only for informing progress towards the SDGs but also for guiding a meaningful, targeted, evidence-informed implementation of the CRPD. Future work to strengthen the conclusions here should include additional regionally or national representative implementations of the MDS, including implementations after any significant disability policies are implemented in order to monitor changes over time.

## Data Availability

Data is available from the corresponding author upon request.

## Abbreviations

*WHO*: World Health Organization
*SDG*: Sustainable Development Goals
*UN*: United Nations
*MDS*: Model Disability Survey
*ICF*: International Classification of Functioning, Disability, and Health
*CRPD*: Convention of the Rights of Persons with Disabilities
*OOB*: Out-of-bag observations
*VIM*: Variable importance measure
*RMSE*: Root mean square error

## References

[1] Ki-Moon B (2014). The road to dignity by 2030: ending poverty, transforming all lives and protecting the planet. Synthesis report of the Secretary-General on the post-2015 sustainable development agenda.

[2] UN Economic and Social Council, Report on the forty-ninth session, Statistical Commission. 2018, United Nations: New York.

[3] International Classification of Functioning, Disability, and Health : Children & Youth version. 2007, Geneva: World Health Organization.

[4] Cieza A, Sabariego C, Bickenbach J, Chatterji S (2018). Rethinking Disability. BMC Med.

[5] Ray M (2017). Functioning and disability in recent research from Cameroon: a narrative synthesis. Pan Afr Med J.

[6] Service d’Appui au Développement Rural (SADER) and P.N.d.D.p. (PNDP) (2015). Plan communal de développement (PCD) de la commune de Bankim.

[7] Bostan C, Oberhauser C, Stucki G, Bickenbach J, Cieza A (2015). Which environmental factors are associated with lived health when controlling for biological health? - a multilevel analysis. BMC Public Health.

[8] Simo Fotso A, Duthe G, Odimegwu C. A comparative analysis of disability measures in Cameroonian surveys. Popul Health Metr. 2019;17(1):16. 10.1186/s12963-019-0198-4 PMC6896774 31805957

[9] Masters GN, Wright BD. In: van der Linden WJ, Hambleton RK, editors. The Partial Credit Model, in Handbook of Modern Item Response Theory. Springer New York, New York; 1997. p. 101–21.

[10] Andrich D, Marais I (2019). A course in Rasch measurement theory : measuring in the educational, social and health sciences. Texts in education.

[11] Sabariego C, et al. Measuring functioning and disability using household surveys: Metric properties of the brief version of the WHO and World Bank Model Disability Survey: Preparation; 2021. 10.1186/s13690-021-00654-9 PMC8273944 34253263

[12] Breiman L (2001). Random forests. Mach Learn.

[13] Core Team R (2016). R: A language and environment for statistical computing.

[14] Strobl C, Boulesteix AL, Zeileis A, Hothorn T (2007). Bias in random forest variable importance measures: illustrations, sources and a solution. BMC Bioinformatics.

[15] Kuhn M (2020). caret: Classification and Regression Training.

[16] Loidl V, Oberhauser C, Ballert C, Coenen M, Cieza A, Sabariego C. Which environmental factors have the highest impact on the performance of people experiencing difficulties in capacity? Int J Environ Res Public Health. 2016;13(4):416. 10.3390/ijerph13040416. 10.3390/ijerph13040416 PMC4847078 27077872

[17] Kamenov K, Cabello M, Ballert CS, Cieza A, Chatterji S, Rojas D, Cerón G, Bickenbach J, Ayuso-Mateos JL, Sabariego C (2018). What makes the difference in people's lives when they have a mental disorder?. Int J Public Health.

[18] World report on disability. 2011, Geneva, Switzerland: World Health Organization. 26131540

[19] United Nations Department of Economic Social Affairs. Disability and Development Report 2018: Realizing the Sustainable Development Goals by, for and with Persons with Disabilities, ed. UN. New York; 2019.

[20] WHO. Supporting the Use of Research Evidence. Available from: https://www.who.int/evidence/sure/en/.

[21] Peters, D.H.a., N.T.a. Tran, and T.a. Adam (2013). Implementation research in health : a practical guide.

